# Chromatin accessibility analysis identifies the transcription factor ETV5 as a suppressor of adipose tissue macrophage activation in obesity

**DOI:** 10.1038/s41419-021-04308-0

**Published:** 2021-10-29

**Authors:** Ren-Dong Hu, Wen Zhang, Liang Li, Zu-Qi Zuo, Min Ma, Jin-Fen Ma, Ting-Ting Yin, Cai-Yue Gao, Shu-Han Yang, Zhi-Bin Zhao, Zi-Jun Li, Gui-Bin Qiao, Zhe-Xiong Lian, Kun Qu

**Affiliations:** 1 Chronic Disease Laboratory, School of Medicine, South China University of Technology, Guangzhou, Guangdong China; 2 Institute of Artificial Intelligence, Hefei Comprehensive National Science Center, Hefei, Anhui China; 3 Guangdong Provincial People’s Hospital, Guangdong Academy of Medical Sciences, Guangzhou, Guangdong China; 4 Department of General Surgery, Guangzhou Digestive Disease Center, Guangzhou First People’s Hospital, the Second Affiliated Hospital of South China University of Technology, Guangzhou, Guangdong China; 5 Guangdong Provincial Institute of Geriatrics, Concord Medical Center, Guangdong Provincial People’s Hospital, Guangdong Academy of Medical Sciences, Guangzhou, Guangdong China; 6 Department of Thoracic Surgery, Guangdong Provincial People’s Hospital, Guangdong Academy of Medical Sciences, Guangzhou, Guangdong China; 7 Bioland Laboratory (Guangzhou Regenerative Medicine and Health Guangdong Laboratory), Guangzhou, Guangdong China

**Keywords:** Obesity, Chronic inflammation

## Abstract

Activation of adipose tissue macrophages (ATMs) contributes to chronic inflammation and insulin resistance in obesity. However, the transcriptional regulatory machinery involved in ATM activation during the development of obesity is not fully understood. Here, we profiled the chromatin accessibility of blood monocytes and ATMs from obese and lean mice using assay for transposase-accessible chromatin sequencing (ATAC-seq). We found that monocytes and ATMs from obese and lean mice exhibited distinct chromatin accessibility status. There are distinct regulatory elements that are specifically associated with monocyte or ATM activation in obesity. We also discovered several transcription factors that may regulate monocyte and ATM activation in obese mice, specifically a predicted transcription factor named ETS translocation variant 5 (ETV5). The expression of ETV5 was significantly decreased in ATMs from obese mice and its downregulation was mediated by palmitate stimulation. The decrease in ETV5 expression resulted in macrophage activation. Our results also indicate that ETV5 suppresses endoplasmic reticulum (ER) stress and *Il6* expression in macrophages. Our work delineates the changes in chromatin accessibility in monocytes and ATMs during obesity, and identifies ETV5 as a critical transcription factor suppressing ATM activation, suggesting its potential use as a therapeutic target in obesity-related chronic inflammation.

## Introduction

Obesity induces a low-grade inflammatory state that contributes to insulin resistance, diabetes, and metabolic syndrome [1, 2]. Excessive adipose tissue expansion results in adipocyte death, hypoxia, and metabolic stress, which initiates and sustains the inflammatory response. Chronic inflammation in adipose tissue is characterized by increased infiltration and activation of innate and adaptive immune cells [3, 4]. Among these immune cells, adipose tissue macrophages (ATMs) are the most abundant cell type, and it has been suggested that they play critical roles in obesity-associated inflammation [5, 6].

ATMs are derived from the bone marrow and infiltrate into the adipose tissue during the development of obesity [7, 8]. Monocytes and macrophages are recruited from the blood and other tissues by hypertrophic adipocytes through the monocyte chemotactic protein-1 (MCP-1)/C-C chemokine receptor type 2 (CCR2) signaling pathway upon the accumulation of extracellular free fatty acids (FFAs) in adipose tissue [9, 10]. The accumulated FFAs include saturated fatty acids (e.g., palmitic acid and stearic acid), mono-unsaturated fatty acids (e.g., oleic acid), and polyunsaturated fatty acids (e.g., linoleic acid) [11]. FFAs can activate the Toll-like receptor 4 (TLR4) signaling pathway, leading to the activation of various protein kinase C (PKCs), endoplasmic reticulum (ER) stress and reactive oxygen species (ROS) generation, which trigger an inflammatory response in macrophages [12]. In adipose tissue from obese mice, ATMs are the main source of inflammatory mediators, such as IL-6 and tumor necrosis factor-α (TNF-α), which can induce insulin resistance by inhibiting the tyrosine phosphorylation of insulin receptor substrate (IRS) [5, 6, 13].

Although infiltrated ATMs in obese mice overexpress *Il6* and *Nos2* [14], recent studies have proposed that they exhibit a metabolically activated phenotype that is distinct from the classical activated phenotype, referred to as metabolically-activated macrophage (MMe) [15]. MMes can be induced in vitro by high glucose plus insulin and palmitate–administration, which are characteristic features of metabolic syndrome [15]. Thus, the lipid-rich environment in adipose tissue stimulates a specific signaling pathway that results in ATM activation in obesity.

While several transcriptional, epigenetic, and metabolic processes involved in macrophage activation have been reported [16], the signaling pathways and transcription factors involved in ATM activation still require further investigation. Accumulating evidence show that the dynamics of chromatin accessibility play important role in altering monocyte/macrophage phenotypes in response to environmental stimuli [17]. Although several regulatory elements, which can control specific gene expression programs in ATMs when triggered by macrophage-polarizing stimuli, have been identified [18, 19], much less is known about the transcriptional changes for regulating ATM differentiation and activation.

In this study, we profiled the chromatin accessibility of blood monocytes and ATMs from lean and obese mice and characterized the regulatory elements associated with macrophage activation during obesity. Particularly, we identified a critical transcription factor (TF), ETS translocation variant 5 (ETV5), which regulates the activation of ATMs. Our findings indicate that it can be a potential therapeutic target in obesity.

## Material and methods

### Cell lines

The murine fibroblast cell line L929, the human embryonic kidney cell line 293 T, and the mouse macrophage line RAW264.7 were purchased from ATCC (American Type Culture Collection). All cells were cultured in DMEM (SH30022.01, Hyclone) medium supplemented with 10% fetal bovine serum (FBS) (FB15015, Clark), 100 U/mL penicillin and 100 µg/mL streptomycin (SV30010, Hyclone) and placed in a cell culture chamber at 37 °C in a humidified atmosphere with 5% CO_2_. Logarithmic growth phase cells were used in all experiments. The supernatant of L929 cell cultures was collected, filtered through a 0.22 μm filter, and stored at −20 °C.

### Mouse strains

C57BL/6 WT mice were purchased from Shanghai Laboratory Animal Center and housed in individually ventilated cages under specific pathogen-free conditions in the Laboratory Animal Research Center, South China University of Technology. All mice were maintained at a 12 h light: 12 h dark cycle with unlimited access to food and water unless indicated. Mice were randomly grouped and investigators were blinded for analysis in each experiment. Sample size was selected based on previously published studies [20, 21]. Six-week old male mice were fed a chow diet (CD, 3.8 kcal/g, energy ratio: 15% protein, 75% carbohydrate, 10% fat, TP24570C) or high-fat diet (HFD containing 7% lactose, 5 kcal/g, energy ratio: 20% protein, 23% carbohydrate, 57% fat, TP24570) (Trophic Animal Feed High-Tech Co., Ltd, China) for 12–14 weeks. The main components of the diets are casein, egg white, cysteine, beef tallow, vegetable oil, lactose, sucrose, fiber, vitamins, minerals, choline, and tertiary butylhydroquinone. 6 to 12-week old C57BL/6 mice were used for bone marrow cell isolation and bone marrow derived macrophage (BMDM) induction. This study was performed in accordance with the principles and guidelines of the National Council for the Control of Animal Experimentation and approved by the Ethics Committee on Animal Use at the School of Medicine, South China University of Technology.

### Glucose tolerance test and insulin tolerance test

For the insulin tolerance test (ITT), mice were fasted for 4 h before intraperitoneal administration of 0.5 U/kg insulin. Blood glucose levels were measured at 0, 15, 30, 60, and 90 min after injection. For the glucose tolerance test (GTT), mice were fasted for 16 h before intraperitoneal administration of 2 g/kg glucose. Blood glucose levels were measured at 0, 15, 30, 60, and 120 min after injection.

### ATM/monocyte isolation and flow cytometry

In each experiment, epididymal and visceral white adipose tissue samples were collected, mixed, and washed with ice-cold phosphate buffer saline (PBS). Adipose tissue samples were minced into small pieces with scissors, suspended in pre-warmed digestion buffer containing 1 mg/mL collagenase II (C6885, Sigma-Aldrich), 1% BSA, and 10 mM HEPES, and incubated at 37 °C for 40 min with vigorous shaking. DMEM containing 2 mM EDTA and 5% FBS was added to stop the digestion. The cell suspension was filtered through a 150-µm nylon filter and centrifuged at 450 × *g* for 5 min at 4 °C. Adipocytes and supernatant were discarded, and red blood cells were depleted using red blood cell lysis buffer (C3702-500 ml, Beyotime, China). Mouse peripheral blood mononuclear cells were isolated using lymphocyte isolation solution (density: 1.081 g/mL). Finally, cells were blocked with purified anti-CD16/32 antibody (BioLegend, San Diego, CA, USA) and stained with the following antibodies: FITC-CD45.2, APC-F4/80, PerCP/Cy5.5-Ly6C, PE-CD11c, APC/Cy7-Ly6G (BioLegend), and PE-CF594-CD11b (BD Biosciences, USA). Flow cytometry data were acquired using a BD LSRFortessa^TM^ Flow Cytometer and analyzed with FlowJo software (BD Biosciences). For cell sorting, the CD11b^+^Ly6Chigh monocytes and CD11b^+^F4/80^+^ ATMs were sorted with a BD FACSAria^TM^ III sorter (BD Biosciences).

### Assay for transposase-accessible chromatin sequencing (ATAC-seq) and data analysis

Assay for transposase-accessible chromatin sequencing (ATAC-seq) libraries of monocytes and macrophages were constructed as previously reported [22]. The data were analyzed using ATAC-pipe [23]. Briefly, we used several parameters to evaluate data quality, including transcription stat site (TSS) enrichment score (reads enriched at ±2 kb around TSS versus background) and read length distribution. Peak calling was performed using MACS2. The number of raw reads mapped to each peak in each condition was quantified using the intersectBed function in BedTools. Raw counts in peaks were normalized using the DESeq package in R. Peak intensity was defined as the log2 of the normalized counts. Peaks with a mean count > 8, fold change (FC)>1.5, and *p* value < 0.05 were compared between CD and HFD conditions. In order to obtain the differential regions between monocytes and macrophages in the HFD condition, the peaks between monocytes and macrophages in CD and HFD groups were compared separately. And, the regions with LogFC_HFD_ – LogFC_CD_>2×*log2 (1.5) were selected. Finally, 9,461 differential peaks were obtained. Unsupervised clustering was performed using Cluster 3.0 and visualized in Treeview. GO and other enriched functions of the cis-regulatory regions was performed using GREAT.

### Bone marrow-derived macrophage (BMDM) culture and polarization

Bone marrow was flushed from mouse femurs and tibias and dissociated into a single-cell suspension. After red blood cell depletion, cells were cultured in DMEM supplemented with 10% heat-inactivated FBS and 1% penicillin-streptomycin solution. To induce BMDM differentiation, 10 ng/mL macrophage colony-stimulating factor (M-CSF) (416-ML, R&D Systems) or 15% L929-conditioned media were added every 2 days for 7 days [24]. BMDMs were stimulated with 100 ng/mL LPS (L4391, Sigma) and 20 ng/mL IFN-γ (315-05, PeproTech) to induce M1 polarization, or 10 ng/mL IL-4 (214-14, PeproTech) and 10 ng/mL IL-13 (413-ML, R&D Systems) to induce M2 polarization. For MMe activation, BMDMs or RAW264.7 cells were treated with 0.4 mM palmitate, 10 nM insulin, and 30 mM glucose for 24 h [25].

### Lentivirus production, infection, and siRNA knockdown

293 T cells were transfected with the plasmids pSIN-GFP (overexpression plasmids), pLKO (shRNA plasmids), pCMV-DR8.9, and pCMV-VSV-G via polybrene (TR-1003, Sigma-Aldrich) following the manufacturer’s instructions. Supernatants containing virus particles were collected 72 hours post transfection, centrifuged at 800 × *g* for 10 min, filtered through a 0.22 µm filter, and stored at −80 °C. siRNAs were purchased from GenePharma (Shanghai, China). Raw264.7 cells or BMDMs were cultured in 6-well plates overnight to achieve 70–80% cell density. For siRNA transfection, 400 µL opti-MEM (Thermo Fisher Scientific, USA) containing 2 µg siRNA or control siRNA and 8uL Turobofect (R0534, TurboFect™ Transfection Reagent, Thermo Fisher Scientific) were added by drop to cell culture. Gene or protein expression levels were detected 24 or 48 hours after infection. For lentivirus infection of Raw264.7 cells, medium were replaced with 1 mL viral particle containing medium after cells reach 70–80% density, and cultured for 6 h. Cells were then digested and transferred to a 6 cm petri dish for further 48-h culture. Meanwhile, the culture medium was replaced with the medium containing 3 µg/mL puromycin (Thermo Fisher Scientific). During cell passage, the concentration of puromycin was slowly adjusted to 5 µg/mL puromycin. Gene or protein expression levels were then analyzed. The sequences of shRNA, siRNA, and primers are listed in Table 1.

**Table 1:** shRNA/overexpression/siRNA/RT-qPCR target sequences.

Knockdown/overexpression Gene	Sequence 5′-3′
Etv5-shRNA-1:	CCGGACAACTATTGTGCCTACGATACTCGAGTATCGTAGGCACAATAGTTGTTTTTTGAATT.
Etv5-shRNA-2:	CCGGGCGACCTTTGATTGACAGAAACTCGAGTTTCTGTCAATCAAAGGTCGCTTTTTGAATT.
Etv5-shRNA-3:	CCGGCAGTCT GATAACTTGGTGCTTCTCGAGAAGCACCAAGTTATCAGACTGTTTTTGAATT.
Overexpression-Etv5-F:	ACGAGCTGTACAAGACTAGTATGGATGGGTTTTGTGATCAGCAAG.
Overexpression-Etv5-R:	CCCTAAATGCATGCGGATCCTTAGTAAGCGAAGCCTTCGGTGTAG.
Etv5-siRNA-1 sense primer:	CCAUCAGAAUUCCCUAUUUTT.
Etv5-siRNA-1 antisense primer:	AAAUAGGGAAUUCUGAUGGTT.
Etv5-siRNA-2 sense primer:	GCCAAAGAUGAUGCCUGAATT.
Etv5-siRNA-2 antisense primer:	UUCAGGCAUCAUCUUUGGCTT.
Etv5-siRNA-3 sense primer:	GCCAUGAAGGAUUCCCGUATT.
Etv5-siRNA-3 antisense primer:	UACGGGAAUCCUUCAUGGCTT.
Hprt-F	AGGTTGCAAGCTTGCTGGT
Hprt-R	TGAAGTACTCATTATAGTCAAGGGCA
Etv5-F	TCAGTCTGATAACTTGGTGCTTC
Etv5-R	GGCTTCCTATCGTAGGCACAA
Arg-1-F	CTCCAAGCCAAAGTCCTTAGAG
Arg-1-R	AGGAGCTGTCATTAGGGACATC
Cd36-F	ATGGGCTGTGATCGGAACTG
Cd36-R	GTCTTCCCAATAAGCATGTCTCC
Abca1-F	AAAACCGCAGACATCCTTCAG
Abca1-R	CATACCGAAACTCGTTCACCC
Fizz1-F	CCAATCCAGCTAACTATCCCTCC
Fizz1-R	ACCCAGTAGCAGTCATCCCA
Tnfa-F	CCCTCACACTCAGATCATCTTCT
Tnfa-R	GCTACGACGTGGGCTACAG
H-2-F	GGACCCCACAGGACTTCACATACT
H-2-R	GCCGTCTTCTCCTTGTTGCTGTGG
Plin2-F	GACCTTGTGTCCTCCGCTTAT
Plin2-R	CAACCGCAATTTGTGGCTC

### RNA isolation and RT-qPCR

Total RNA from cultured cells or ATMs was isolated using either TRIzol Reagent (15596018, Invitrogen) or RNeasy MinElute Cleanup Kit (74204, QIAGEN). First-strand cDNA was synthesized and subjected to qPCR using SYBR Green on a LightCycler 96 System (Roche, Switerland). Gene expression was normalized to GAPDH, β-actin, or Hprt using the ΔΔCt method.

### Western blot

Cells were lysed using 1X RIPA lysis and extraction buffer (89901, Thermo Fisher Scientific). Total protein was determined using a BCA Protein Assay kit (23227, Thermo Fisher Scientific). For western blot analysis, the supernatant after lysis was added to an equal volume of loading buffer and heated to 95 °C for 10 min. Equal amounts of proteins (20–2000 µg/mL) were separated using a 12% SDS-PAGE and transferred onto PVDF membranes. The membranes were blocked with 10% skimmed milk and incubated overnight at 4 °C with a monoclonal anti-ETV5 antibody (MA5-15646, Thermo Fisher Scientific; 1: 1,000) or monoclonal anti-β-actin antibody (A5441, Sigma-Aldrich; 1: 5,000). After washing, the membranes were incubated with a secondary antibody (7076 s, Cell Signaling Technology; 1: 2,000) at room temperature for 1 h. Images were collected using a Tanon-5200 Chemiluminescent Imaging System (Tanon, China). Densitometric analyses were performed using Image J (National Institutes of Health). The experiments were repeated three times on separate days.

### RNA-sequencing and data analysis

RNA quality was determined using an Agilent 2100 Bioanalyzer. Sequencing libraries were prepared using the Illumina Truseq^TM^ RNA sample prep Kit v2. Pearson correlation analysis of the total gene expression matrix was performed using R software (version 4.0). The Limma R package was used to identify the differentially expressed genes. Heat map schemes and volcano plots were created using the corresponding R package. The genes that were upregulated (log2fc > 2) in the Raw264.7/sh-Etv5 compared with control Raw264.7 cells were selected to perform Gene Ontology (GO) analysis using the clusterProfiler package, and the top 20 enriched GO terms were listed. The Gene Set Enrichment Analysis (GSEA) was performed in R using gene sets that were download from Mouse Genome Informatics.

### Single-cell RNA-sequencing data analysis

Single-cell RNA-sequencing data of the stromal vascular fraction from mouse inguinal white adipose tissue (iWAT) were obtained from the GEO database (accession number GSE154047). Data from wild-type mice were used for the analysis. Data were analyzed using R software (Version 4.0). Genes that were expressed in less than five cells and cells with less than 200 or greater than 6000 genes were discarded. Dimensionality reduction analysis was performed using the Seurat R package (version 4.0). Principal component analysis (PCA) was performed using the variable genes and determined significant principal components based on the ElbowPlot function from the Seurat package. The top 30 PCs were selected for t-distributed stochastic neighbor embedding (tSNE) analysis. Myeloid cell cluster with feature *Lyz2* expression was chosen for further analysis.

### ELISA assay for IL-6

Supernatants were collected after macrophage culture and IL-6 protein concentrations were measured using a mouse IL-6 ELISA kit (E-MSEL-M0001, Elabscience, China) according to the manufactories’ instructions.

### Statistical analysis

A two-tailed unpaired Student’s t-test was conducted in GraphPad Prism 5 (GraphPad Software, San Diego, CA, USA) to determine statistical significance. Different test methods were used to analyze body weight change, GTT and ITT result in HFD- and CD-fed mice. We first checked data distribution and equal variances by Shapiro-Wilk test and levene test, respectively. We analyzed body weight change and GTT result in HFD- and CD-fed mice using two-way ANOVA with repeated measurements and Holm–Sidak post hoc test. We used Mann-Whitney U test to analyze the ITT result. All results were obtained from at least two independent experiments. Error bars represent mean ± standard deviation.

## Results

### Landscape of chromatin accessibility of peripheral blood monocytes and adipose tissue macrophages in lean and obese mice

We established a murine model of obesity by feeding mice with a HFD. Mice fed with a CD were considered as lean mice. Compared with CD-fed mice, HFD-fed mice showed increased body weight (Fig. s1A). ITT and GTT results indicated that HFD-fed mice develop insulin resistance and glucose intolerance (Fig. s1B, C). Moreover, ATMs from HFD-fed mice contain a higher percentage of the CD11c^+^ population (Fig. s1D, E), indicating an activated phenotype, in line with previous studies [14, 26, 27].

**Fig. 1:**
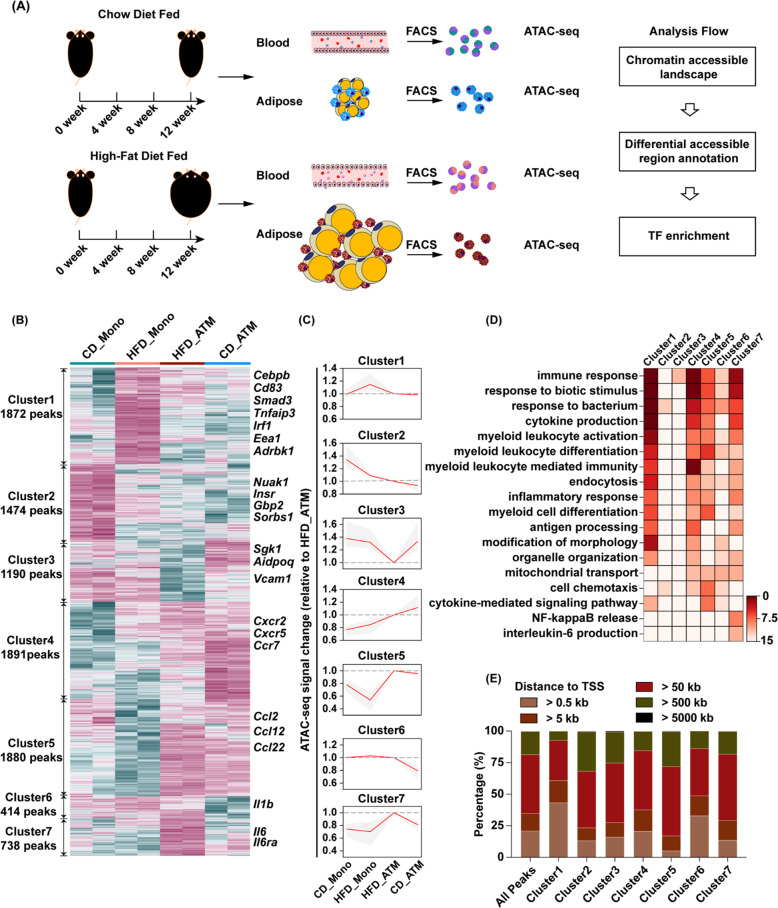
Landscape of chromatin accessibility in peripheral blood monocytes and adipose tissue macrophages. **A** The ATAC library construction and sequencing processes of peripheral blood monocytes and adipose tissue macrophages pooled from CD (*n* = 9) or HFD (*n* = 6) -fed mice. **B** Heatmap showing seven clusters of distinctive chromatin accessibility patterns between CD_Mono, HFD_Mono, HFD_ATM, and CD_ATM with representative genes annotated of each cluster. **C** Line plots showing mean (red line) and S.D. (gray ribbon) of mean-centered normalized log2 values for each cluster. **D** Heatmap of top 18 most enriched biological processes of seven clusters shown as -log10 raw binomial *p* values, as calculated by Genomic Regions Enrichment of Annotations Tool (GREAT); **E** Distribution of distance to nearest TSS of genes from different gene clusters.

We then isolated the peripheral blood monocytes and ATMs from both HFD- and CD-fed mice, and performed ATAC-seq library construction and sequencing (Fig. 1A). TSS enrichment analysis and aligned fragment length distribution of all samples indicated high data quality (Fig. s2A, B). Correlation analysis of all replicates indicated excellent reproducibility between replicates (Fig. s2C). We found that the *Itgam* gene locus was more accessible in ATMs, while *Ly6c2* and *Ccr2* gene loci were more accessible in monocytes (Fig. s2D). Consistent with previous reports that *Ccr2* is highly expressed in blood monocytes from HFD-fed mice [9], we observed high chromatin dynamics in *Ccr2* gene locus in monocytes. Similarly, we found that the promoter of pro-inflammatory macrophage marker *Nos2* was also more accessible in ATMs isolated from HFD-fed mice (Fig. s2D). Next, we performed PCA of the 8 samples and found that within each group, the same cell type from obese (HFD_Mono, HFD_ATM) and lean mice (CD_Mono, CD_ATM) could also be clustered together (Fig. s2E).

We then compared the differential regulatory elements between HFD_ATM and HFD_Mono, relative to CD_ATM and CD_Mono (Fig. s2F). We discovered 9,461 differential chromatin-accessible regions, and classified them into seven distinct clusters (C1-C7) using unsupervised hierarchical clustering (Fig. 1B, C). We annotated the functions of these seven clusters and found that the functions associated with immune activation were enriched in five clusters (C1, C3, C4, C6, and C7) (Fig. 1D). C1 was composed of elements that are associated with immune activation and myeloid leukocyte differentiation and are more accessible in monocytes from HFD-fed mice (HFD_Mono) than in other cells, suggesting that these regions are associated with monocyte activation. Moreover, we found that nearly half of the elements in C1 were located at the proximal end of the TSS (Fig. 1E). The proximal regions in C1 were related to gene transcription and nucleotide bioavailability (Fig. s3A), whereas the distal regions were mainly related to immune activation (Fig. s3B). C4 and C5 were enriched in elements associated with immune activation, cell differentiation, and cell chemotaxis, suggesting that they may be associated with inflammatory monocyte activation and migration to adipose tissue. C6 and C7 were more accessible in HFD_ATM and strongly enriched in functions related to cell chemotaxis, cytokine-mediated signaling, NF-κB signaling pathway and IL-6 production. Altogether, our data demonstrate that monocytes and ATMs possess different chromatin accessibility landscapes in obese and lean states. In addition, we identified several regulatory elements that are associated with monocyte and macrophage activation during obesity.

### Transcription factor occupancy network during monocyte and ATM activation

**Fig. 2:**
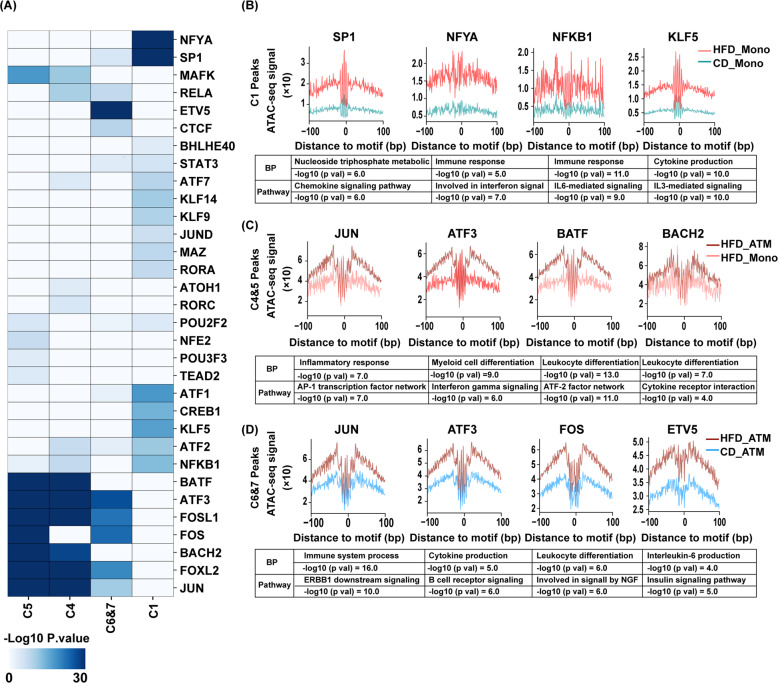
Transcription factor occupancy network during monocyte and ATM activation in CD- and HFD-fed mice. **A** Enriched motifs of transcript factors in different cluster peaks with *p* values estimated with homer4.8. **B** Visualization of ATAC-seq footprint motifs and GREAT annotation in cluster modules of HFD_Mono vs CD_Mono, **C** HFD_ATM vs. HFD_Mono, and **D** HFD_ATM vs. CD_ATM.

To explore the TFs involved in the regulation of monocyte and macrophage activation during obesity, we predicted TFs that can bind to accessible sites in the C1, C4, C5, C6, and C7 regions (Fig. 2A). We identified several TFs that are enriched in different regulatory element clusters, which may participate in monocyte activation (C1), monocyte to macrophage differentiation (C4 and C5), and macrophage activation (C6 and C7) in obesity.

We first focused on the TFs associated with monocyte activation. SP1, a critical factor for monocyte-specific expression of CD14 and inflammatory response against pathogens [28], was enriched in the C1 regions. Additionally, NFYA, NFKB1, and KLF5 were enriched in the C1 regions (Fig. 2A, B). NFYA activates the transcription of genes highly expressed in mature monocytes, while NFKB1 and KLF5 mediate inflammatory cytokine production in monocytes [29, 30]. We also performed TF footprint analysis to provide evidence of the direct occupancy of TF candidates on genomic DNA. We found that SP1, NFYA, NFKB1, and KLF5 were most likely physically bonded to the chromatin-accessible sites, which were more accessible in monocytes from HFD-fed mice (Fig. 2B).

Obesity is associated with increased monocyte migration into the adipose tissue and differentiation into ATMs [9, 10]. We then focused on TFs that regulate monocyte differentiation to ATMs. JUN and BATF were enriched at accessible sites in C4 and C5 (Fig. 2C). JUN and BATF have been previously reported to regulate precursor cell differentiation into macrophages [31, 32]. Interestingly, we found that BACH2 and ATF3, which are reported to suppress the differentiation and activation of macrophages [33, 34], were also enriched at accessible sites in C4 and C5 (Fig. 2C), indicating that the regions that can recruit negative regulators are also accessible during the differentiation and activation of macrophages.

Finally, we focused on TFs that regulate ATM activation. JUN, ATF3 FOS, and ETV5 were enriched at accessible sites in C4 and C5 (Fig. 2D), among which FOS has been reported to mediate macrophage activation [35]. ETV5 was enriched at accessible sites in C6 and C7. Although the function of ETV5 in macrophages has not been described yet, this result indicates that it could be a potential regulator of ATM activation. Through GREAT annotation of biological process, we found that ETV5 binding elements were associated with IL-6 production (Fig. 2D), indicating that it might regulate ATM-mediated chronic inflammation in obese adipose tissue.

### ETV5 downregulation in metabolically activated macrophages

**Fig. 3:**
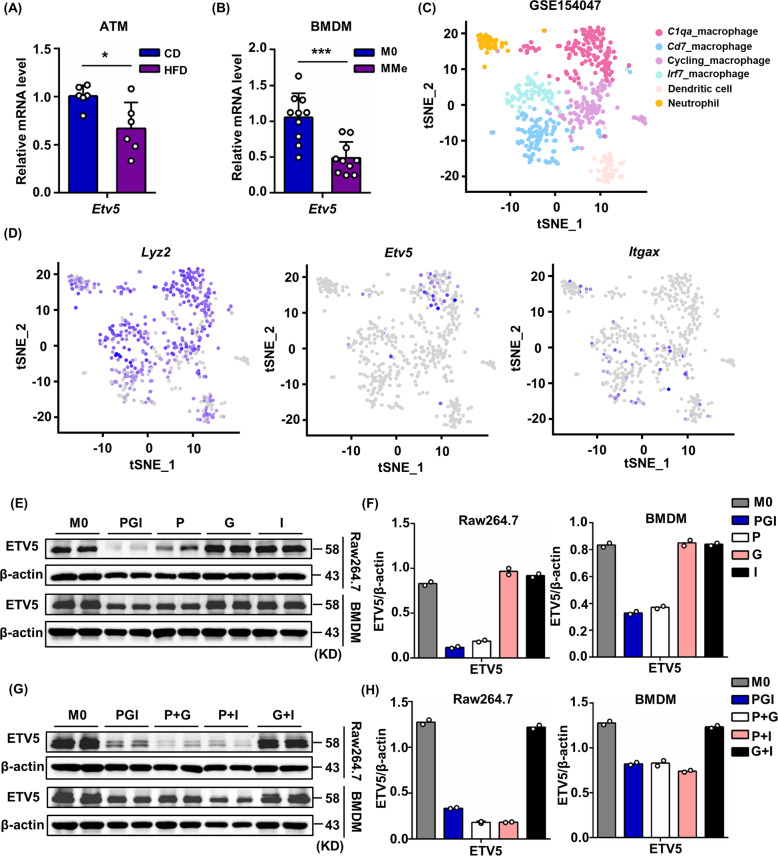
ETV5 expression was downregulated in ATMs from HFD-fed mice and in in vitro induced MMe. **A** *Etv5* mRNA levels in ATMs from CD- (*n* = 6) and HFD-fed (*n* = 6) mice. **B** *Etv5* mRNA levels in BMDMs after 24 h of stimulation with palmitate (P, 0.4 mM), glucose (G, 30 mM), and insulin (I, 10 nM). Gene expression levels were assessed by RT-qPCR. Data were pooled from two independent experiments. **C** Single-cell RNA-sequencing data of stromal vascular fraction from mouse iWAT. tSNE plot showing myeloid cell clusters. **D** Featureplot showing *Lyz2*, *Etv5* and *Itgax* expression by different myeloid cell clusters. **E** ETV5 protein levels and **F** quantification in Raw264.7 cells and BMDMs after treatment with P, G, I alone or in combination for 24 h. **G** ETV5 protein levels and **H** quantification in Raw264.7 cells and BMDMs after treatment with P, G, and I in pairs for 24 h. Data in **E** and **G** were representative of three independent experiments. *p < 0.05, **p < 0.01, ***p < 0.001, Student’s t test.

The transcription factor ETV5 has been reported to regulate insulin exocytosis and hepatic fatty acid metabolism [36, 37], but its role in macrophage activation remains unknown. Therefore, we aimed to determine whether ETV5 can regulate macrophage activation in obesity. The expression of *Etv5* was decreased in the ATMs of HFD-fed mice (Fig. 3A). As ATMs from obese mice are biased towards a MMe phenotype, we analyzed *Etv5* expression in MMe induced in vitro (Fig. s4B), and found that the expression of *Etv5* was also decreased (Fig. 3B). We also induced M1 (Fig. s4C) and M2 (Fig. s4D) polarization of BMDMs and Raw264.7 cells in vitro and found that ETV5 expression did not change under M1 or M2 polarization conditions (Fig. s4E), indicating that ETV5 is specifically involved in the metabolic activation of macrophages.

To investigate the heterogeneity in the expression of *Etv5* in different macrophage subpopulations, we analyzed single-cell RNA-sequencing data of the stromal vascular fraction from mouse iWAT (GEO accession number GSE154047) [38]. Six myeloid cell clusters were identified, each of which was named according to their featured gene expression (Fig. s4A). We found four macrophage clusters (*C1qa*_macrophage, *Cd7*_macropahge, Cycling_macrophage, *Irf7*_macrophage), one neutrophil cluster and one dendritic cell cluster (Fig. 3C). *C1qa*_macrophages expressed high levels of *Retnla a*nd *Selenop*, which are genes associated with alternative macrophage activation (Fig. s4A). They also expressed high level of *Etv5*. Interestingly, macrophages with high *Itgax* expression exhibited low *Etv5* expression, indicating that activated ATMs present a decreased *Etv5* expression in vivo (Fig. 3D). To induce MMe in vitro, we treated macrophages with palmitate, glucose, insulin, or a combination of these compounds. We found that ETV5 was downregulated only in the presence of palmitate (Fig. 3E–H). These results suggest that the FFAs may be the main factors to induce ETV5 downregulation in macrophages.

### Knockdown of *Etv5* induces expression of genes associated with inflammation response and IL-6 production in macrophages

**Fig. 4:**
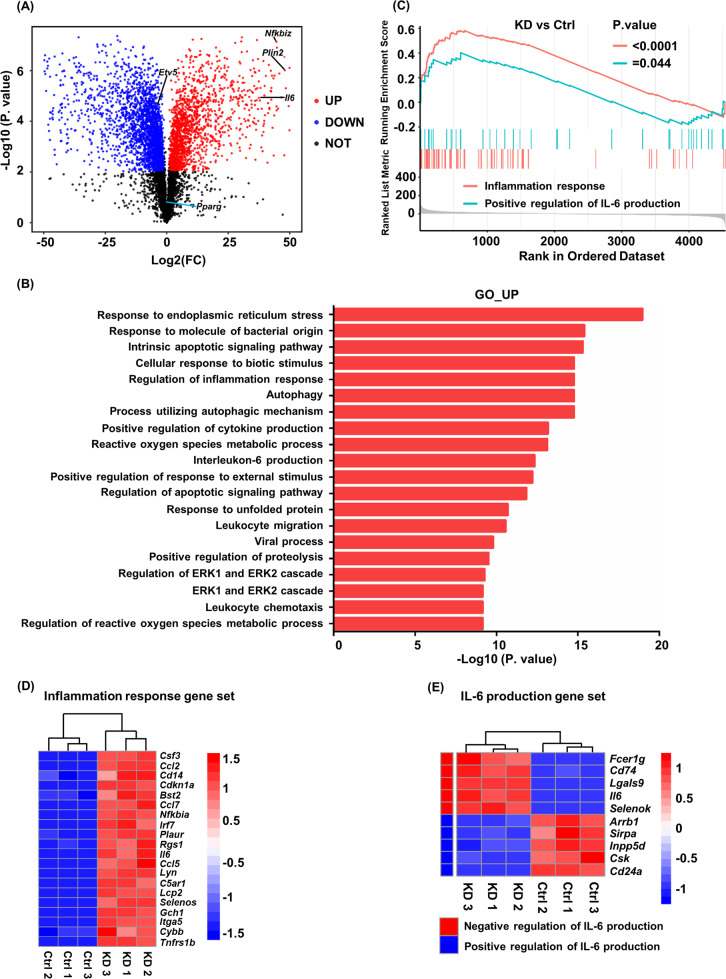
Knockdown *Etv5* induces expression of genes associated with macrophage activation and IL-6 production. **A** RNA-seq analysis of upregulated and downregulate genes after knockdown of *Etv5* in Raw264.7 cells. **B** GO analysis showing top20 enriched biological functional pathways of upregulated genes in Raw264.7/sh-*Etv5* cells**. C** GSEA analysis of inflammation response pathway (red) and positive regulation of IL-6 production pathway (blue) after knockdown of *Etv5* in Raw264.7 cells. **D** Heatmap showing genes that were upregulated in inflammatory response gene set after knockdown of *Etv5* in Raw264.7 cells. **E** Heatmap showing genes that were positive regulated (red) and negative regulated (blue) in IL-6 production gene set after knockdown of *Etv5* in Raw264.7 cells.

To identify genes regulated by ETV5 in macrophages, we knocked down the expression of *Etv5* by shRNA in Raw264.7 cells, and then performed RNA-sequencing of stably transfected cells. We identified 2,017 upregulated genes and 2,525 downregulated genes in Raw264.7/sh-*Etv5* cells, compared with Raw264.7/sh-NC cells (Fig. 4A). We found that the knockdown of *Etv5* in macrophages induced the upregulation of *Il6* and *Plin2*, which are MMe markers (Fig. 4A). We also found increased expression of *Nfkbiz* (Fig. 4A), which encodes IκB-ζ, an essential factor for the activation of a subset of inflammatory genes including IL-6 [39]. These results indicated that macrophages were activated when *Etv5* expression was reduced.

We then sought to identify pathways that mediate macrophage activation in Raw264.7/sh-*Etv5* cells. PPAR-γ has been reported to control ATM alternative activation and improve insulin resistance [40], but its expression did not change after *Etv5* silencing, indicating that there are other pathways regulated by ETV5 (Fig. 4A). GO functional analysis showed that upregulated genes in Raw264.7/sh-*Etv5* were enriched in pathways associated with ER stress, inflammatory response, and IL-6 production (Fig. 4B). We focused on the IL-6 production pathway since IL-6 is critical in mediating chronic inflammation in obese adipose tissue. In addition, our ATAC-seq data indicated that ETV5 is involved in the inflammatory response and IL-6 production (Fig. 2C). We then performed GSEA analysis of the differentially expressed genes and found that the pathways related to the inflammatory response (*p* value < 0.001) and positive regulation of IL-6 production pathway were significantly enriched (*p* value = 0.044) (Fig. 4C) in Raw264.7/sh-*Etv5* cells. In addition, heat maps showed that *Il6* gene ranked 11^th^ in inflammation response gene set and 4^th^ in IL-6 production gene set (Fig. 4D, E). These results suggest that ETV5 negatively regulates the inflammatory response and IL-6 production pathways, and knock down of *Etv5* induces MMe features in macrophages.

### ETV5 negatively regulates *Il6* expression in macrophages

**Fig. 5:**
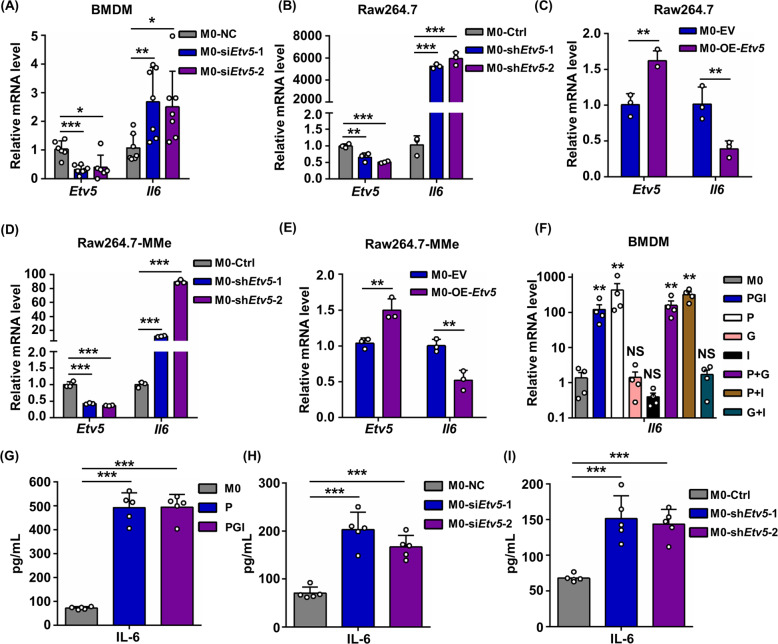
The expression of *Il6* after knockdown or overexpression of *Etv5* in macrophages. **A** BMDMs were transfected with siRNA negative control (siRNA NC) or siRNA specific to *Etv5* (siRNA *Etv5*) 48 h and *Il6* expression was assessed by RT-qPCR 48 h later. *Il6* expression levels of Raw264.7 cells after knockdown of *Etv5* using shRNA **B** or overexpression of *Etv5* **C**. *Il6* expression levels of Raw264.7 cells after knockdown of *Etv5* using shRNA **D** or overexpression of *Etv5* **E** under MMe induction condition. **F** *Il6* expression levels of BMDMs after treatment with palmitate, glucose, and insulin alone or with a combination of these compounds for 24 h. **G–I** Measuring IL-6 protein levels in the supernatant of Raw264.7 cell culture by ELISA. Cells were treated with palmitate (P) or PGI **G**, or underwent Etv5 knockdown using siRNA **H**, or shRNA **I**. Data in this result were all representative of three independent experiments. *p < 0.05, **p < 0.01, ***p < 0.001, Student’s t test.

To confirm that ETV5 negatively regulates the production of IL-6 in macrophages, we analyzed *Il6* expression levels in Raw264.7 cells and BMDMs after silencing or overexpressing ETV5. Knockdown of ETV5 in BMDMs or Raw264.7 cells (Fig. s4F) augmented the expression of *Il6* (Fig. 5A, B), whereas ETV5 overexpression in the Raw264.7 cell line (Fig. s4F) resulted in a significant decrease in *Il6* expression (Fig. 5C). To better simulate the in vivo obese microenvironment, we treated Raw264.7 cells in which ETV5 was silenced or overexpressed with palmitate, glucose, and insulin to induce MMe activation, and observed that the expression of *Il6* was negatively regulated by *Etv5* in MMe macrophages (Fig. 5D, E). Additionally, we treated macrophages with palmitate, glucose, insulin, or combinations of these compounds, and found that *Il6* expression was upregulated only in the presence of palmitate (Fig. 5F). We also detected the IL-6 protein level in the supernatant using ELISA. We found that palmitate treatment can induce IL-6 production, and the supplement of glucose and insulin did not promote IL-6 secretion, which is consistent with the mRNA expression (Fig. 5G). Knockdown of *Etv5* using either siRNA or shRNA also promoted the secretion of IL-6 by macrophages (Fig. 5H, I). These results demonstrate that the FFAs in the microenvironment of obese adipose tissue may induce IL-6 production of ATMs by downregulating ETV5 (Fig. s5).

## Discussion

Transcriptional regulation of macrophage activation is currently a subject of great interest. To date, the regulation of macrophage activation has largely been attributed to a small group of transcription factors including NF-κB, AP-1, HIFs, STATs, and PPARs. Our observations introduce a novel regulator, ETV5 to this process and more specifically, identify that ETV5 acts as a critical factor in regulating IL-6 expression in MMe. Thus, targeting ETV5 in macrophages may be a suitable strategy for the treatment of obesity and other chronic inflammatory diseases.

Previously, a genome-wide association study has indicated that ETV5 is positively correlated with weight and body mass index in humans [41]. In addition, ETV5 was reported to be able to control insulin secretion by β cells in the pancreas [37]. *Etv5*-knockout mice present impaired insulin secretion, which results in severe glucose intolerance and insulin resistance [37]. A recent study showed that upon glucose stimulation or HFD-feeding, CRL4^COP1^ E3 ligase can promote ETV5 degradation in pancreatic islet cells, thus mediating insulin over-secretion and promote insulin resistance and obesity [42]. Our work suggests that glucose intolerance and insulin resistance in *Etv5*-knockout mice may also be attributed to the excessive activation of ATMs. Moreover, ETV5 regulates fatty acid metabolism in hepatocytes by promoting PPARγ expression [36]. However, knockdown of *Etv5* did not change the expression of *Pparg* in macrophages under our experimental conditions. Thus, ETV5 may play different roles in ATMs and adipocytes during the development of obesity.

During adipose tissue growth, adipocyte death and hypoxia, FFAs and inflammatory cytokines promote macrophage activation and cytokine production [43–45]. A recent study reported that, in obesity, adipose tissue-resident macrophages adopt specific metabolic programs characterized by the activation of various metabolic pathways, including oxidative phosphorylation and glycolysis [46]. In particular, glycolysis contributes to increased inflammatory cytokine production. Inhibition of glycolysis by 2-deoxy-D-glucose can reduce both lactate production and glucose oxidation by macrophages in adipose tissue from obese mice [46]. When adipose tissue expands and creates a hypoxic microenvironment, hypoxia inducible factor (HIF)-1α is activated to promote glycolysis by inducing the expression of enzymes in the glycolysis pathway. However, recent evidence has suggested that adipose tissue inflammation does not require HIF-1α-mediated signaling [46] but relies on HIF-2α instead [47]. The inhibition of HIF-2α markedly augments the palmitate-induced expression of pro-inflammatory genes in adipocytes, and high-fat diet exacerbates adipose tissue inflammation in HIF-2α deficient mice [47]. Our results suggest that ETV5 is associated with the HIF-1α signaling pathway. It has been reported that IL-4/IL-13 stimulation can upregulate the *Etv5* expression levels in macrophages [48]. We speculate that the inflammation caused by hypoxia in adipose tissue is closely related to ETV5, but the mechanism requires further investigation.

The recruitment of monocytes to adipose tissue and their subsequent differentiation into macrophages is a characteristic of adipose tissue subjected to chronic inflammation in obesity [9, 10]. We also profiled chromosome accessibility during the activation of monocytes and their differentiation into macrophages, and analyzed gene regulatory elements and predicted transcription factors that may regulate these processes, which may also provide other targets against monocytes for obesity therapy. However, monocytes and ATMs are heterogeneous populations, single-cell analyses are required in the future to further delineate the cell-to-cell heterogeneity and regulatory dynamics of monocyte activation, their differentiation into ATMs, and ATM activation during obesity.

Previous studies have reported that ETV5 can regulate T cell cytokine production. ETV5 can directly promote the transcription of *Il10* [49], and recruit histone-modifying enzymes to promote *Il17* [50] and *Il9* [51] expression. However, further investigation is still needed to fully uncover the mechanism of how ETV5 regulate macrophage activation. In this study, we found that knockdown of *Etv5* promoted the expression of *NFKBIZ* and activated the *ERK1/2* pathway in macrophages. Further evidence suggests that genes encode IL-6 are under the control of transcriptional nuclear factor-kappa B (NF-κB) [52, 53]. NFKBIZ, a unique member of the IκB family with a distinct role as a coactivator of NF-κB, has been reported to increase IL-6 production in BMDMs [39, 54, 55]. Activation of the ERK1/2 and NF-κB pathways cause the release of pro-inflammatory cytokines such as IL-6, IL-8, and TNF-α, which could lead to an inflammatory response [56]. Moreover, we observed that macrophages with decreased *Etv5* expression upregulated genes associated with cellular response to ER stress, which can induce the activation of NF-κB pathway. We speculated that ETV5 in macrophages mediates the inflammatory response through negative regulation of ERK/NF-κB pathway, but the mechanism requires further investigation.

Finally, RNA-seq results showed that the transcription factor ETV5 is associated with the IL-6 production pathway and inflammatory response in macrophages, indicating that the function of ETV5 in regulating inflammation may not be limited to ATMs in obesity. Thus, ETV5 may be a promising therapeutic target in other inflammatory diseases.

## Supplementary information

Supplementary material

Supplementary figure 1

Supplementary figure 2

Supplementary figure 3

Supplementary figure 4

Supplementary figure 5

Reproducibility Checklist

## Data Availability

RNA-seq data files were deposited into the Gene Expression Omnibus (GEO) database under the accession number GSE166294. Other datasets used and/or analyzed during the current study are available from the corresponding author on reasonable request.

## References

[1] Glass CK, Olefsky JM (2012). Inflammation and lipid signaling in the etiology of insulin resistance. Cell Metab.

[2] Tsalamandris S, Antonopoulos AS, Oikonomou E, Papamikroulis GA, Vogiatzi G, Papaioannou S (2019). The role of inflammation in diabetes: current concepts and future perspectives. Eur Cardiol.

[3] de Luca C, Olefsky JM (2008). Inflammation and insulin resistance. FEBS Lett.

[4] Sun M, Zheng S, Gao X, Lin Z (2020). The role of immune cells in obesogenic memory. Cell Mol Immunol.

[5] Fain JN (2006). Release of interleukins and other inflammatory cytokines by human adipose tissue is enhanced in obesity and primarily due to the nonfat cells. Vitam Horm.

[6] Zeyda M, Stulnig TM (2007). Adipose tissue macrophages. Immunol Lett.

[7] Weisberg SP, McCann D, Desai M, Rosenbaum M, Leibel RL, Ferrante AJ (2003). Obesity is associated with macrophage accumulation in adipose tissue. J Clin Invest.

[8] Xu H, Barnes GT, Yang Q, Tan G, Yang D, Chou CJ (2003). Chronic inflammation in fat plays a crucial role in the development of obesity-related insulin resistance. J Clin Invest.

[9] Oh DY, Morinaga H, Talukdar S, Bae EJ, Olefsky JM (2012). Increased macrophage migration into adipose tissue in obese mice. Diabetes.

[10] Engin AB (2017). Adipocyte-macrophage cross-talk in obesity. Adv Exp Med Biol.

[11] Feng R, Luo C, Li C, Du S, Okekunle AP, Li Y (2017). Free fatty acids profile among lean, overweight and obese non-alcoholic fatty liver disease patients: a case - control study. Lipids Health Dis.

[12] Korbecki J, Bajdak-Rusinek K (2019). The effect of palmitic acid on inflammatory response in macrophages: an overview of molecular mechanisms. Inflamm Res.

[13] Prada PO, Ropelle ER, Mourao RH, de Souza CT, Pauli JR, Cintra DE (2017). Statement of Retraction. EGFR tyrosine kinase inhibitor (PD153035) improves glucose tolerance and insulin action in high-fat diet-fed mice. Diabetes.

[14] Lumeng CN, Bodzin JL, Saltiel AR (2007). Obesity induces a phenotypic switch in adipose tissue macrophage polarization. J Clin Invest.

[15] Kratz M, Coats BR, Hisert KB, Hagman D, Mutskov V, Peris E (2014). Metabolic dysfunction drives a mechanistically distinct proinflammatory phenotype in adipose tissue macrophages. CELL METAB.

[16] Chen S, Yang J, Wei Y, Wei X (2020). Epigenetic regulation of macrophages: from homeostasis maintenance to host defense. Cell Mol Immunol.

[17] Davis FM, Gallagher KA (2019). Epigenetic mechanisms in monocytes/macrophages regulate inflammation in cardiometabolic and vascular disease. Arterioscler Thromb Vasc Biol.

[18] Horvath A, Daniel B, Szeles L, Cuaranta-Monroy I, Czimmerer Z, Ozgyin L (2019). Labelled regulatory elements are pervasive features of the macrophage genome and are dynamically utilized by classical and alternative polarization signals. Nucleic Acids Res.

[19] Tang MS, Miraldi ER, Girgis NM, Bonneau RA, Loke P (2020). Alternative activation of macrophages is accompanied by chromatin remodeling associated with lineage-dependent DNA shape features flanking PU.1 motifs. J Immunol.

[20] Kozuka C, Yabiku K, Sunagawa S, Ueda R, Taira S, Ohshiro H (2012). Brown rice and its component, gamma-oryzanol, attenuate the preference for high-fat diet by decreasing hypothalamic endoplasmic reticulum stress in mice. Diabetes.

[21] Vila IK, Badin PM, Marques MA, Monbrun L, Lefort C, Mir L (2014). Immune cell Toll-like receptor 4 mediates the development of obesity- and endotoxemia-associated adipose tissue fibrosis. Cell Rep.

[22] Buenrostro JD, Wu B, Chang HY, Greenleaf WJ (2015). ATAC-seq: a method for assaying chromatin accessibility genome-wide. Curr Protoc Mol Biol.

[23] Zuo Z, Jin Y, Zhang W, Lu Y, Li B, Qu K (2019). ATAC-pipe: general analysis of genome-wide chromatin accessibility. Brief Bioinform.

[24] Lacey DC, Achuthan A, Fleetwood AJ, Dinh H, Roiniotis J, Scholz GM (2012). Defining GM-CSF- and macrophage-CSF-dependent macrophage responses by in vitro models. J Immunol.

[25] Coats BR, Schoenfelt KQ, Barbosa-Lorenzi VC, Peris E, Cui C, Hoffman A (2017). Metabolically activated adipose tissue macrophages perform detrimental and beneficial functions during diet-induced obesity. Cell Rep.

[26] Cho KW, Morris DL, DelProposto JL, Geletka L, Zamarron B, Martinez-Santibanez G (2014). An MHC II-dependent activation loop between adipose tissue macrophages and CD4+ T cells controls obesity-induced inflammation. Cell Rep.

[27] Patsouris D, Li PP, Thapar D, Chapman J, Olefsky JM, Neels JG (2008). Ablation of CD11c-positive cells normalizes insulin sensitivity in obese insulin resistant animals. Cell Metab.

[28] Zhang DE, Hetherington CJ, Tan S, Dziennis SE, Gonzalez DA, Chen HM (1994). Sp1 is a critical factor for the monocytic specific expression of human CD14. J Biol Chem.

[29] Marziali G, Perrotti E, Ilari R, Coccia EM, Mantovani R, Testa U (1999). The activity of the CCAAT-box binding factor NF-Y is modulated through the regulated expression of its A subunit during monocyte to macrophage differentiation: regulation of tissue-specific genes through a ubiquitous transcription factor. Blood.

[30] Chen HL, Chong IW, Lee YC, Tsai JR, Yuan SS, Wang HM (2014). Kruppel-like factor 5 mediates proinflammatory cytokine expression in lipopolysaccharide-induced acute lung injury through upregulation of nuclear factor-kappaB phosphorylation in vitro and in vivo. Mediators Inflamm.

[31] Gaynor R, Simon K, Koeffler P (1991). Expression of c-jun during macrophage differentiation of HL-60 cells. Blood.

[32] Liao J, Humphrey SE, Poston S, Taparowsky EJ (2011). Batf promotes growth arrest and terminal differentiation of mouse myeloid leukemia cells. Mol Cancer Res.

[33] Itoh-Nakadai A, Matsumoto M, Kato H, Sasaki J, Uehara Y, Sato Y (2017). A Bach2-Cebp gene regulatory network for the commitment of multipotent hematopoietic progenitors. Cell Rep.

[34] Suganami T, Yuan X, Shimoda Y, Uchio-Yamada K, Nakagawa N, Shirakawa I (2009). Activating transcription factor 3 constitutes a negative feedback mechanism that attenuates saturated Fatty acid/toll-like receptor 4 signaling and macrophage activation in obese adipose tissue. Circ Res.

[35] Higuchi Y, Setoguchi M, Yoshida S, Akizuki S, Yamamoto S (1988). Enhancement of c-fos expression is associated with activated macrophages. Oncogene.

[36] Mao Z, Feng M, Li Z, Zhou M, Xu L, Pan K (2021). ETV5 regulates hepatic fatty acid metabolism through PPAR signaling pathway. Diabetes.

[37] Gutierrez-Aguilar R, Kim DH, Casimir M, Dai XQ, Pfluger PT, Park J (2014). The role of the transcription factor ETV5 in insulin exocytosis. Diabetologia.

[38] Henriques F, Bedard AH, Guilherme A, Kelly M, Chi J, Zhang P (2020). Single-cell RNA profiling reveals adipocyte to macrophage signaling sufficient to enhance thermogenesis. Cell Rep.

[39] Yamamoto M, Yamazaki S, Uematsu S, Sato S, Hemmi H, Hoshino K (2004). Regulation of Toll/IL-1-receptor-mediated gene expression by the inducible nuclear protein IkappaBzeta. Nature.

[40] Odegaard JI, Ricardo-Gonzalez RR, Goforth MH, Morel CR, Subramanian V, Mukundan L (2007). Macrophage-specific PPARgamma controls alternative activation and improves insulin resistance. Nature.

[41] Thorleifsson G, Walters GB, Gudbjartsson DF, Steinthorsdottir V, Sulem P, Helgadottir A (2009). Genome-wide association yields new sequence variants at seven loci that associate with measures of obesity. Nat Genet.

[42] Lin H, Yan Y, Luo Y, So WY, Wei X, Zhang X (2021). IP6-assisted CSN-COP1 competition regulates a CRL4-ETV5 proteolytic checkpoint to safeguard glucose-induced insulin secretion. Nat Commun.

[43] Berg AH, Lin Y, Lisanti MP, Scherer PE (2004). Adipocyte differentiation induces dynamic changes in NF-kappaB expression and activity. Am J Physiol Endocrinol Metab.

[44] Trujillo ME, Scherer PE (2006). Adipose tissue-derived factors: impact on health and disease. Endocr Rev.

[45] Unamuno X, Gomez-Ambrosi J, Ramirez B, Rodriguez A, Becerril S, Valenti V, et al. NLRP3 inflammasome blockade reduces adipose tissue inflammation and extracellular matrix remodeling. Cell Mol Immunol 2019;2019-09-24. 10.1038/s41423-019-0296-z PMC8115140 31551515

[46] Boutens L, Hooiveld GJ, Dhingra S, Cramer RA, Netea MG, Stienstra R (2018). Unique metabolic activation of adipose tissue macrophages in obesity promotes inflammatory responses. Diabetologia.

[47] Choe SS, Shin KC, Ka S, Lee YK, Chun JS, Kim JB (2014). Macrophage HIF-2alpha ameliorates adipose tissue inflammation and insulin resistance in obesity. Diabetes.

[48] Roy S, Schmeier S, Arner E, Alam T, Parihar SP, Ozturk M (2015). Redefining the transcriptional regulatory dynamics of classically and alternatively activated macrophages by deepCAGE transcriptomics. Nucleic Acids Res.

[49] Koh B, Hufford MM, Sun X, Kaplan MH (2017). Etv5 regulates IL-10 production in Th cells. J Immunol.

[50] Pham D, Sehra S, Sun X, Kaplan MH (2014). The transcription factor Etv5 controls TH17 cell development and allergic airway inflammation. J Allergy Clin Immunol.

[51] Koh B, Hufford MM, Pham D, Olson MR, Wu T, Jabeen R (2016). The ETS family transcription factors Etv5 and PU.1 function in parallel to promote Th9 cell development. J Immunol.

[52] Karin M, Greten FR (2005). NF-kappaB: linking inflammation and immunity to cancer development and progression. Nat Rev Immunol.

[53] Brasier AR (2010). The nuclear factor-kappaB-interleukin-6 signalling pathway mediating vascular inflammation. Cardiovasc Res.

[54] Slowikowski K, Nguyen HN, Noss EH, Simmons DP, Mizoguchi F, Watts G (2020). CUX1 and IkappaBzeta (NFKBIZ) mediate the synergistic inflammatory response to TNF and IL-17A in stromal fibroblasts. Proc Natl Acad Sci USA.

[55] Horber S, Hildebrand DG, Lieb WS, Lorscheid S, Hailfinger S, Schulze-Osthoff K (2016). The Atypical Inhibitor of NF-kappaB, IkappaBzeta, Controls Macrophage Interleukin-10 Expression. J Biol Chem.

[56] Park J, Min JS, Kim B, Chae UB, Yun JW, Choi MS (2015). Mitochondrial ROS govern the LPS-induced pro-inflammatory response in microglia cells by regulating MAPK and NF-kappaB pathways. Neurosci Lett.

