# Supplementary material for: Chromatin accessibility analysis identifies the transcription factor ETV5 as a suppressor of adipose tissue macrophage activation in obesity
Source: Cell Death Dis. 2021 Oct 29;12(11):1023. doi: 10.1038/s41419-021-04308-0 (PMC8556336; doi:10.1038/s41419-021-04308-0)
Supplement: Supplementary file 1 — Supplementary material [file 41419_2021_4308_MOESM1_ESM.docx]

**Chromatin accessibility analysis identifies the transcription factor ETV5 as a suppressor of adipose tissue macrophage activation in obesity**

Ren-Dong Hu^1, #^, Wen Zhang^2, #^, Liang Li^3, *^, Zu-Qi Zuo^2^, Min Ma^1^, Jin-Fen Ma^1^, Ting-Ting Yin^4^, Cai-Yue Gao^1^, Shu-Han Yang^1^, Zhi-Bin Zhao^3^, Zi-Jun Li^5^, Gui-Bin Qiao^6^, Zhe-Xiong Lian^1, 7, *^ and Kun Qu^2, *^

1 Chronic Disease Laboratory, School of Medicine, South China University of Technology, Guangzhou, Guangdong, China.

2 Department of Oncology, The First Affiliated Hospital of USTC, Division of Molecular Medicine, Hefei National Laboratory for Physical Sciences at Microscale, Division of Life Sciences and Medicine, University of Science and Technology of China, Hefei, Anhui 230027, China.

3 Guangdong Provincial People's Hospital, Guangdong Academy of Medical Sciences, Guangzhou, China.

4 Department of General Surgery, Guangzhou Digestive Disease Center, Guangzhou First People’s Hospital, the Second Affiliated Hospital of South China University of Technology, Guangzhou, China.

5 Guangdong Provincial Institute of Geriatrics, Concord Medical Center, Guangdong Provincial People's Hospital, Guangdong Academy of Medical Sciences, Guangzhou, Guangdong, China.

6 Department of Thoracic Surgery, Guangdong Provincial People's Hospital, Guangdong Academy of Medical Sciences, Guangzhou, China.

7 Bioland Laboratory (Guangzhou Regenerative Medicine and Health Guangdong Laboratory), 510005 Guangzhou, China.

^#^ Ren-Dong Hu and Wen Zhang contributed equally to this work.

Corresponding authors:

^*^Kun Qu, Department of Oncology, The First Affiliated Hospital of USTC, Division of Molecular Medicine, Hefei National Laboratory for Physical Sciences at Microscale, Division of Life Sciences and Medicine, University of Science and Technology of China, Hefei, Anhui 230027, China. Email: qukun@ustc.edu.cn

^*^Zhe-Xiong Lian, M.D., Ph.D., Chronic Disease Laboratory, School of Medicine, South China University of Technology, Guangzhou, 510006, China. Email: zxlian@scut.edu.cn

^*^Liang Li, Guangdong Provincial People's Hospital, Guangdong Academy of Medical Sciences, Guangzhou, China. Email: lil2009@scut.edu.cn

**Supplementary figure 1. High fat diet induced obesity in mice.** **(A)** Body weight gain of mice fed with HFD (n =7) or CD (n =9). **(B)** Insulin tolerance test (ITT) and **(C)** glucose tolerance test (GTT) of mice after 12 weeks HFD (n =7) or CD (n =9) feeding. **(D, E)** Percentage of CD11c^+^ ATMs in CD- (n =5) and HFD-fed (n =5) mice. Data are expressed as mean ± SD. Two-way ANOVA with repeated measurements and Holm–Sidak post-hoc test **(A-B)**, Mann-Whitney U test **(C)**, Student’s t test **(E)**, **p* < 0.05, ***p* < 0.01, ****p* < 0.001.

**Supplementary figure 2. Quality control of ATAC-seq data.** **(A)** ATAC-seq signal intensity in ±2 kb around TSS. **(B)** Fragment distribution, red line showed the average value of eight samples; **(C)** Correlation of chromatin accessibility between replicates. **(D)** Normalized ATAC-seq profiles of the *Itgam*, *Ly6c2*, *Nos2* and *Ccr2* gene loci at monocytes/ATMs from CD- and HFD-fed mice. **(E)** Principle component analysis (PCA) of the chromatin accessibility for ATAC-seq signal. **(F)** Differential regulatory elements between HFD_ATM vs. HFD_Mono relative to CD_ATM vs. CD_Mono.

**Supplementary figure 3. Biological functions of differential regulatory elements in cluster1.**

**(A-B):** Top 20 most enriched biological process of TSS-proximal **(A)** and TSS-distal accessible regions **(B)** in cluster1 shown as -log10 raw binomial *p* values, as calculated by GREAT.

**Supplementary figure 4. Establishment of M1, M2, and MMe macrophage induction system.** **(A)** The expression of marker genes in different myeloid clusters of mice adipose tissue. **(B)** The expression of MMe macrophage markers *Il6*, *Cd36*, *Abca1* and *Plin2* in *in vitro* induced MMe (n=4). **(C)** The expression of *Nos2*, *H-2* in *in vitro* induced M1 macrophages (n=3) and **(D)** *Fizz1*, *Arg-1* and *Ym-1* in *in vitro* induced M2 macrophages (n=3). **(E)** ETV5 protein levels and quantification in *in vitro* induced M1 and M2 macrophages. **(F)** ETV5 protein levels and quantification in Raw264.7 cells with *Etv5* overexpression and knockdown. Raw264.7 cells were infected with *Etv5*-overexpressed lentivirus (termed as OE-*Etv5*), a negative control (termed as EV), *Etv5* knockdown lentivirus (termed as sh*Etv5*-1, sh*Etv5*-2, sh*Etv5*-3), and a scramble control (termed as NC). Data in **(B)-(F)** are representative of three independent experiments. **p* < 0.05, ***p* < 0.01, ****p* < 0.001, Student’s t test.

**Supplementary figure 5. Working model.** Using ATAC-seq, we profiled chromatin accessibility of monocytes and ATMs from CD- and HFD- fed mice. We found that during the activation of ATMs, FFA-ETV5-IL-6 axis stimulates adipose tissue inflammation, which may be associated with obesity-induced insulin resistance.
