# Supplementary figures and images for: Chromatin accessibility analysis identifies the transcription factor ETV5 as a suppressor of adipose tissue macrophage activation in obesity
Source: Cell Death Dis. 2021 Oct 29;12(11):1023. doi: 10.1038/s41419-021-04308-0 (PMC8556336; doi:10.1038/s41419-021-04308-0)

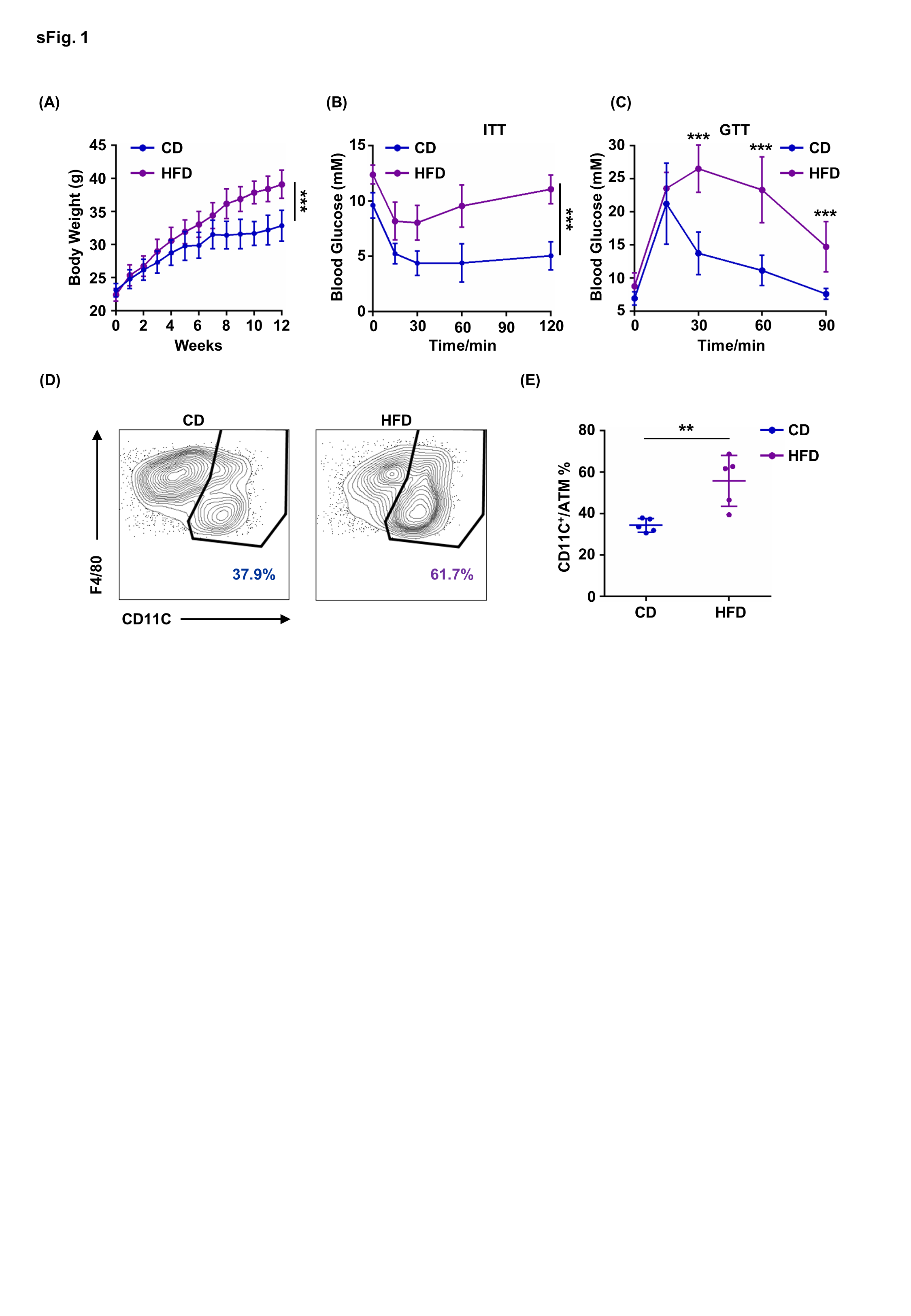

Supplement: Supplementary file 2 — Supplementary figure 1 [file 41419_2021_4308_MOESM2_ESM.tif]

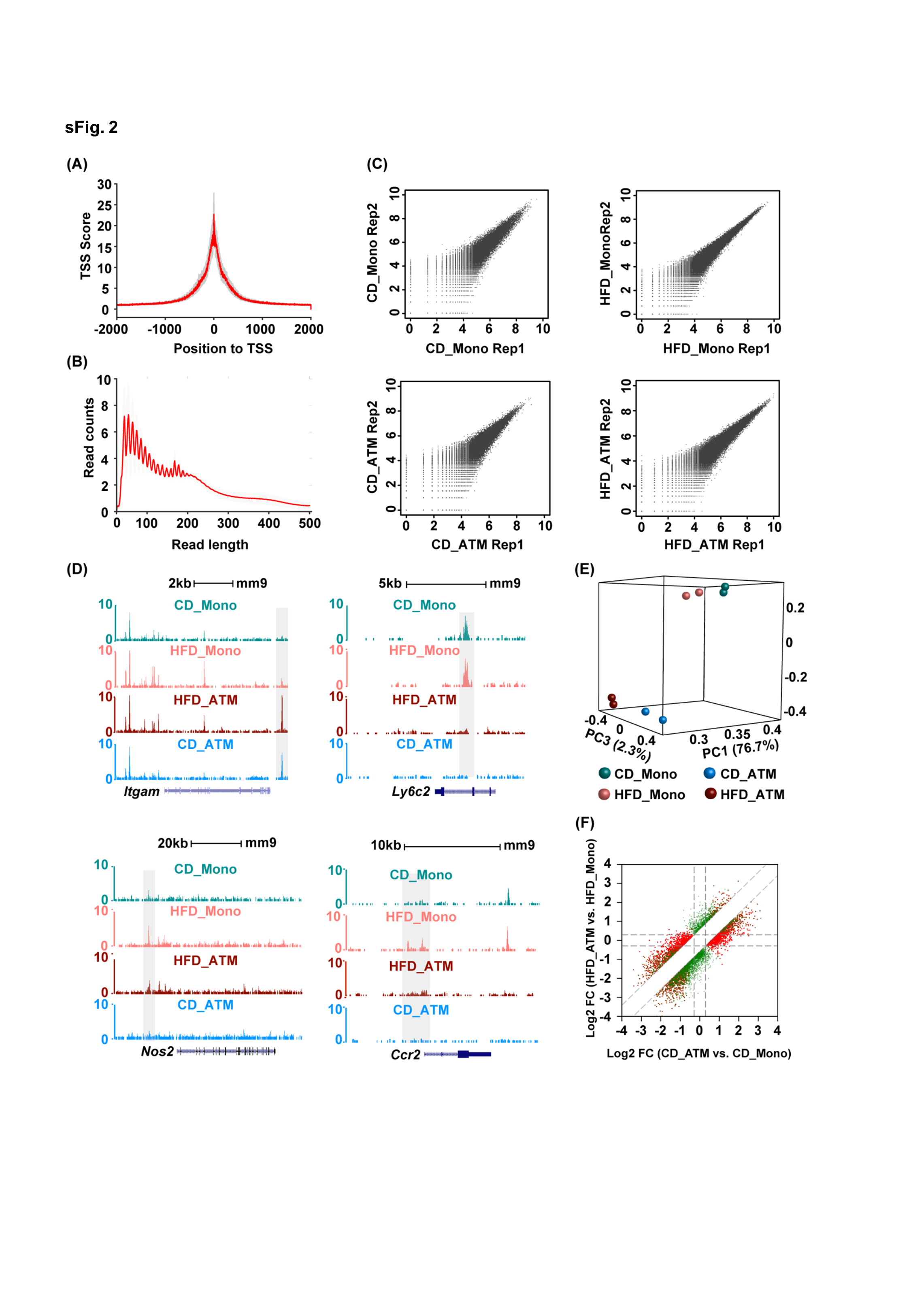

Supplement: Supplementary file 3 — Supplementary figure 2 [file 41419_2021_4308_MOESM3_ESM.tif]

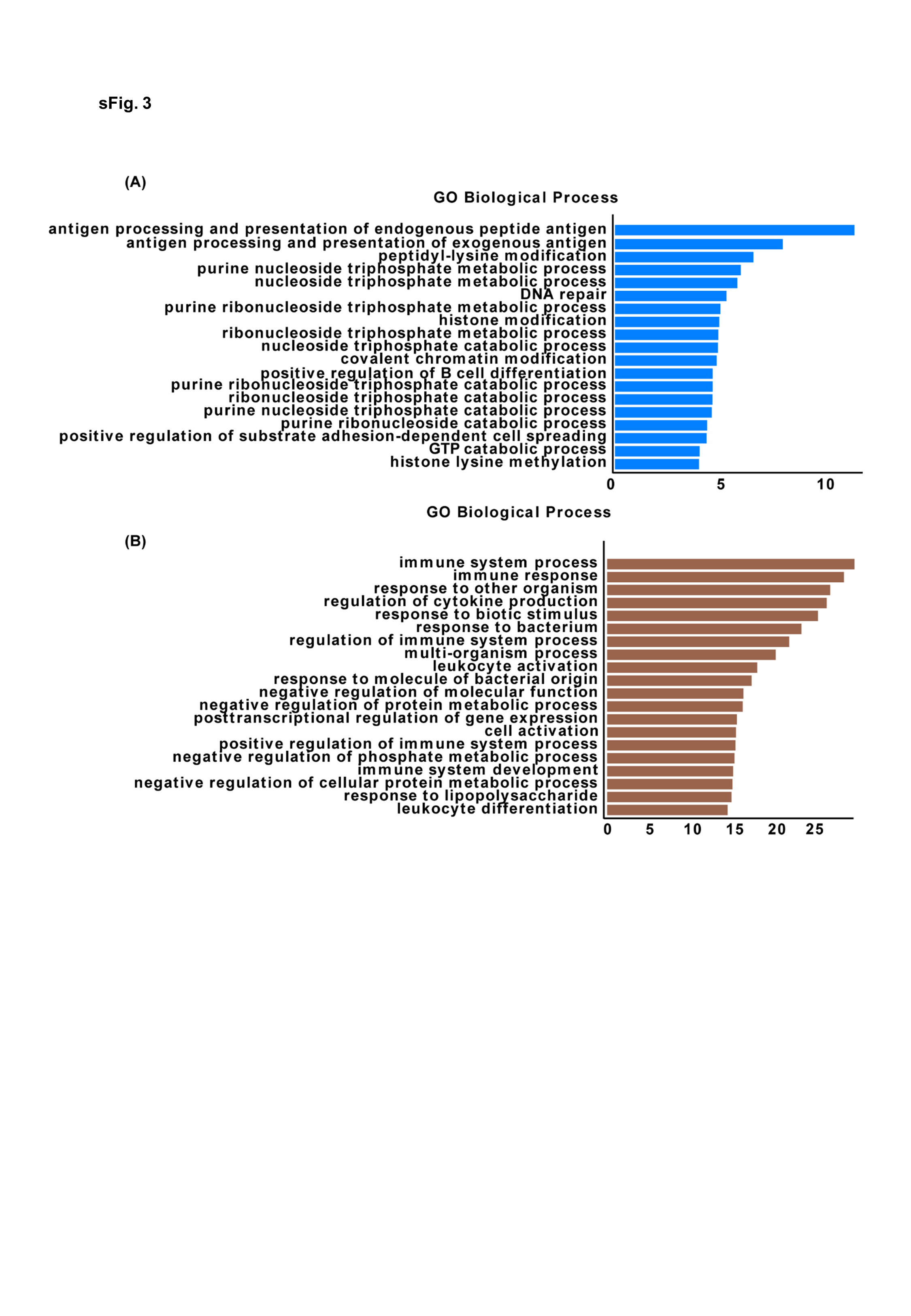

Supplement: Supplementary file 4 — Supplementary figure 3 [file 41419_2021_4308_MOESM4_ESM.tif]

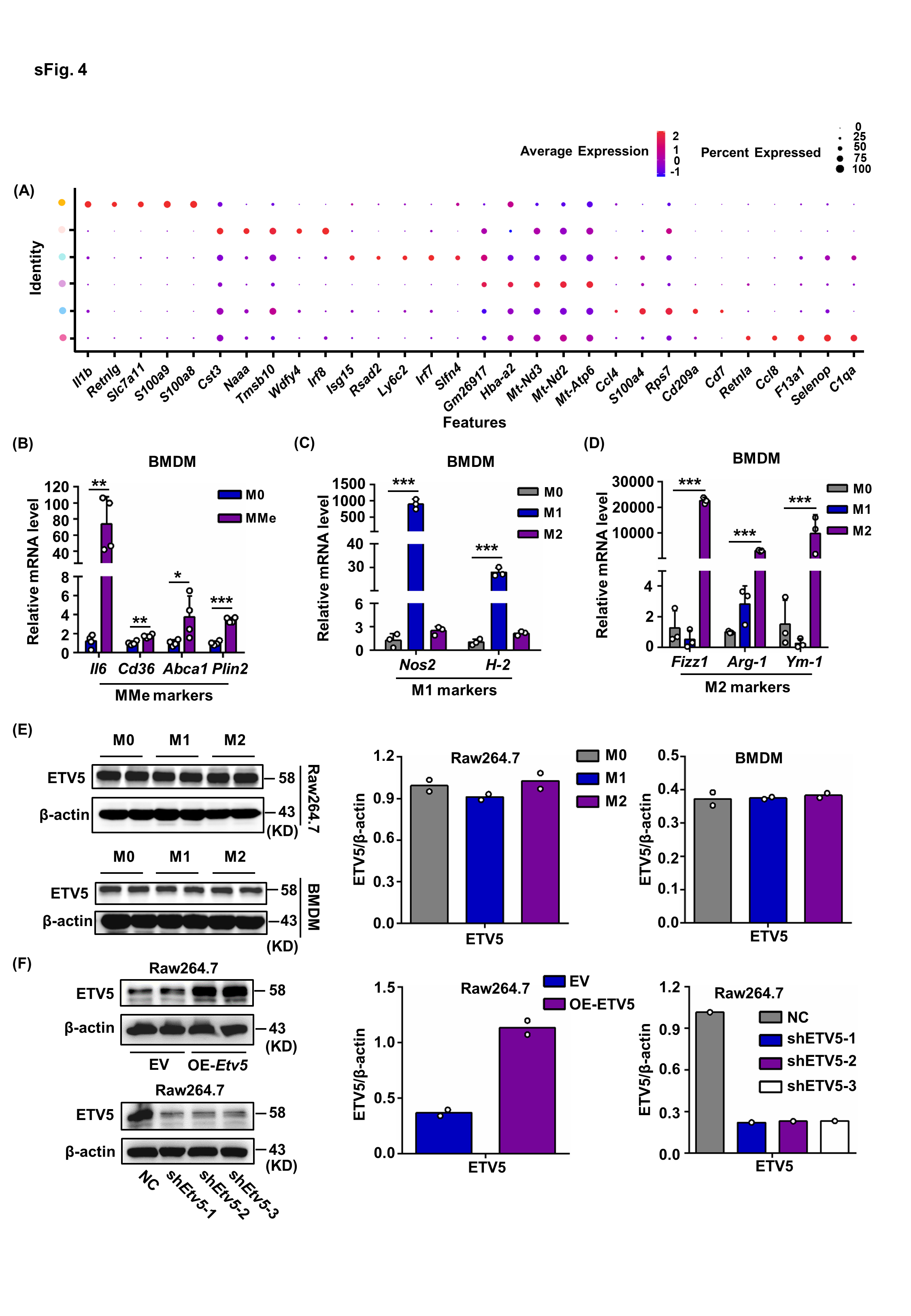

Supplement: Supplementary file 5 — Supplementary figure 4 [file 41419_2021_4308_MOESM5_ESM.tif]

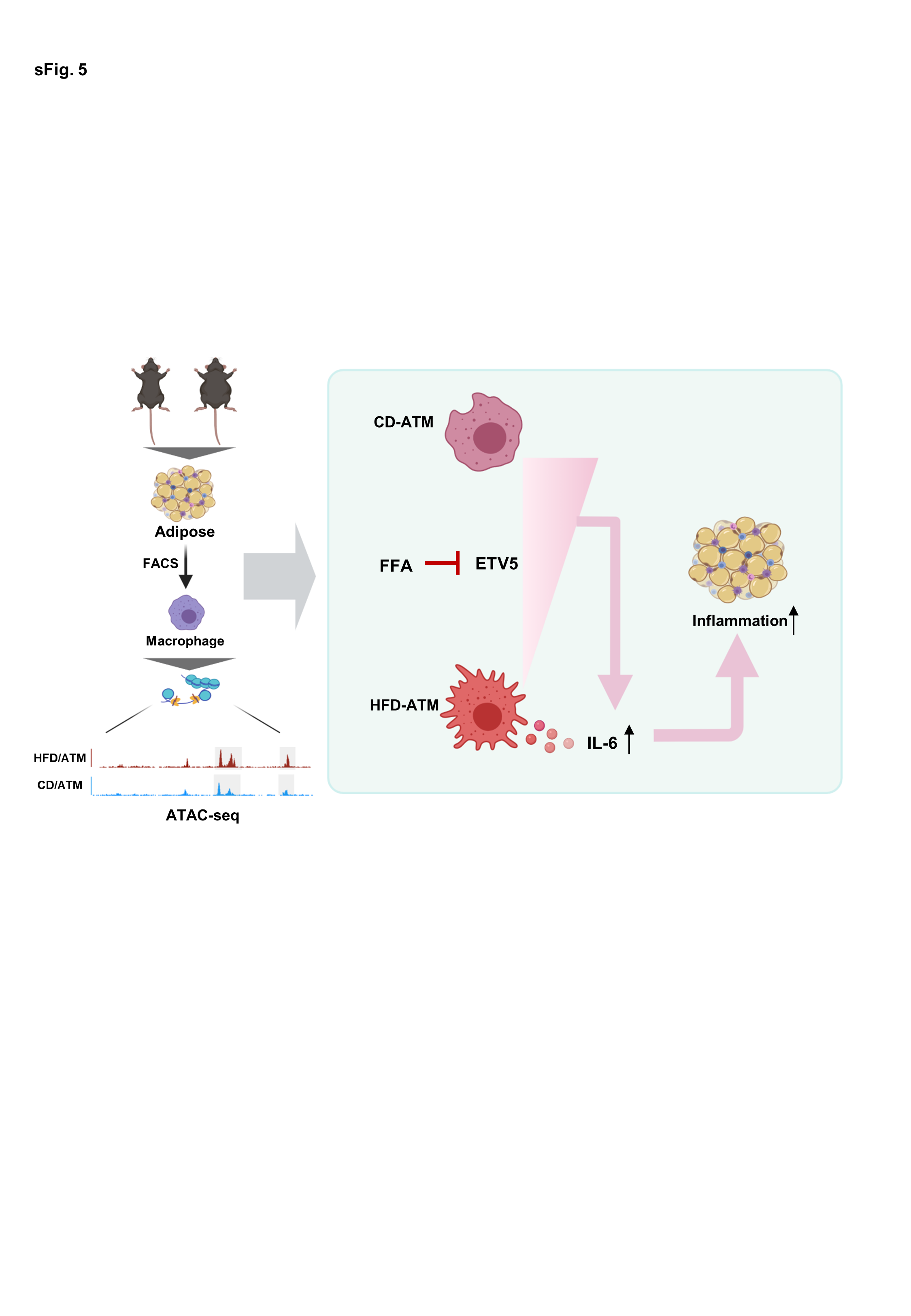

Supplement: Supplementary file 6 — Supplementary figure 5 [file 41419_2021_4308_MOESM6_ESM.tif]
